# Effect of anti-diabetic drugs in dialysis patients with diabetes: a nationwide retrospective cohort study

**DOI:** 10.1186/s12933-021-01364-w

**Published:** 2021-09-08

**Authors:** Shih-Hsiang Ou, Hsin-Yu Chen, Nai-Wen Fang, Chun-Hao Yin, Chien-Liang Chen, Jin-Shuen Chen

**Affiliations:** 1 Division of Nephrology, Department of Internal Medicine, Kaohsiung Veterans General Hospital, Kaohsiung, Taiwan; 2 Faculty of Medicine, School of Medicine, National Yang Ming Chiao Tung University, Taipei, Taiwan; 3 Graduate Institute of Clinical Medicine, Kaohsiung Medical University, Kaohsiung, Taiwan; 4 Department of Pediatrics, Kaohsiung Veterans General Hospital, Kaohsiung, Taiwan; 5 Department of Medical Education and Research, Kaohsiung Veterans General Hospital, Kaohsiung, Taiwan; 6 Faculty of Medicine, School of Medicine, National Defense Medicine, Taipei, Taiwan

**Keywords:** End-stage renal disease, Dialysis, Type 2 diabetes mellitus, Anti-diabetic drugs

## Abstract

**Background:**

Type 2 diabetes mellitus is common in patients undergoing dialysis. However, the association between anti-diabetic drug use and survival outcomes is rarely discussed. We aimed to investigate whether continued anti-diabetic medication use affects the survival of diabetic dialysis patients and whether different hypoglycemic drug use influences prognosis.

**Methods:**

Using a nationwide database, we enrolled patients with incident end-stage renal disease under maintenance dialysis during 2011–2015 into the pre-existing diabetes dialysis (PDD), incident diabetes after dialysis (IDD), and non-diabetic dialysis (NDD) groups. The PDD group was further subclassified into patients who continued (PDD-M) and discontinued (PDD-NM) anti-diabetic drug use after dialysis.

**Results:**

A total of 5249 dialysis patients were examined. The PDD-NM group displayed a significantly higher mortality rate than the IDD, PDD-M, and NDD groups (log-rank test P < 0.001). The PDD-M group had a significantly lower risk of death, regardless of insulin (P < 0.001) or oral hypoglycemic agent (OHA) (P < 0.001) use. Initial insulin administration or OHA had no statistically significant effect on overall mortality in the IDD group. But OHA use had better survival trends than insulin administration for the older (P = 0.02) and male subgroups (P = 0.05).

**Conclusions:**

For dialysis patients with diabetes, continuous administration of anti-diabetic drugs after dialysis and choice of medication may affect outcomes.

**Supplementary Information:**

The online version contains supplementary material available at 10.1186/s12933-021-01364-w.

## Background

The worldwide incidence and prevalence of end-stage renal disease (ESRD) have been increasing annually. Type 2 diabetes mellitus (T2DM) is the leading cause of ESRD in most developed countries and one of the common comorbidities in dialysis patients [1, 2]. Most patients are diagnosed with T2DM before the initiation of dialysis; however, some develop new-onset diabetes afterward. This situation is becoming more common as the survival time of ESRD patients is increasing. Some epidemiologic studies reported an association between pre-existing diabetes in dialysis patients and a poor prognosis [3, 4]. But discussions on clinical outcomes between patients with new-onset diabetes after entering dialysis and other dialysis groups are scarce.

Good glycemic control in diabetic patients is proven to be associated with fewer complications, decreased incidence of cardiovascular disease and mortality, regardless of renal function [5, 6]. In some cases, diabetic patients may experience spontaneous resolution of hyperglycemia with near-normal hemoglobin A1C (HbA1C) when their renal function declines gradually [7]. Such patients are also prone to hypoglycemia after using some glucose-lowering drugs, which may hinder continued medication use when renal failure [8]. The biological significance and clinical implications of this phenomenon are unclear [9]. Currently, few studies have explored the clinical difference and prognosis between patients with continued anti-diabetic medication administration after initiation of dialysis and those without.

According to the Kidney Disease Outcomes Quality Initiative (KDOQI) guideline, only several oral hypoglycemic agents (OHA) kinds can be administered in dialysis patients, including sulfonylurea (SU, such as glipizide and gliclazide), meglitinide (repaglinide), thiazolidinediones (TZD), and dipeptidyl-peptidase IV (DPP-4) inhibitors [10]. Most studies focused only on the impact of a certain anti-diabetic drug in this population; whether the choice of different kinds of OHA or insulin will affect the mortality of dialysis patients in different periods remains unknown.

The purpose of this nationwide retrospective study was to use a large dataset from Taiwan’s National Health Insurance Research Database (NHIRD) to explore whether continuing anti-diabetic medication administration after initiation of dialysis affects the survival of dialysis patients with pre-existing diabetes, and whether administering different hypoglycemic drugs influences prognoses. Additionally, we aimed to investigate the risk factors associated with the mortality of dialysis patients with pre-existing and new-onset diabetes.

## Methods

### Data collection

This observational cohort study used the “Longitudinal Health Insurance Database 2010 (LHID2010)”, established from NHIRD by the Health and Welfare Data Science Center (HWDC) in Taiwan. The Taiwan National Health Insurance (NHI) Program was implemented in 1995, offering comprehensive medical care coverage to more than 99 % of the country’s population of 23 million inhabitants. The LHID2010 contains all the original claims data of 2 million beneficiaries randomly sampled from individuals covered by the NHI Program between January 1 and December 31, 2010. The LHID2010 contains integral information, including demographic data, diagnostic codes, registration files, detailed prescriptions, dates of clinical visits, dates of admission and discharge, and medical expenditures for the enrollees of two million beneficiaries. No statistically significant difference was found in the distributions of age and sex between the cohort in the LHID2010 and the Taiwan NHI enrollees. Several scientific research articles have been published and confirmed the validation of the NHIRD database in the T2DM with ESRD population [11, 12]. The disease and diagnostic codes recorded in the registry of clinical visits and hospital care were based on the International Classification of Diseases, Ninth and Tenth Revision, Clinical Modifications (ICD-9-CM and ICD-10-CM). To ensure patient confidentiality, only data with encoded identification numbers were released. The HWDC approved the application after reviewing all the required medical documents. This study was also approved by the Institutional Review Board of the Kaohsiung Veterans General Hospital (IRB No.: KSVGH18-CT10-07), who supervised the study in accordance with the tenets of the Declaration of Helsinki (1975) and its later amendment (2013). The requirement of informed consent was waived.

### Study design

This observational study enrolled patients who began incident maintenance dialysis (hemodialysis or peritoneal dialysis) between January 1, 2011 and December 31, 2015, through outpatient claims. ESRD patients on maintenance dialysis were defined as having undergone dialysis for more than 90 days. The exclusion criteria included patients who were younger than 20 years; diagnosed with type 1 diabetes mellitus; had undergone maintenance dialysis before tracking; and survived less than 90 days after confirmed maintenance dialysis. The remaining patients were followed up from the day of their first dialysis treatment until their death, dialysis withdrawal, or the end of follow-up (December 31, 2016).

The study participants were classified into patients with T2DM diagnosed before the initiation of dialysis (pre-existing diabetes dialysis group; PDD) and without (non-pre-existing diabetes dialysis group; NPDD). T2DM was defined as having had at least one admission code, or three or more outpatient codes for T2DM (ICD-9: 250.X; ICD-10: E11.X), as well as having received anti-diabetic drugs for at least 90 days in the last year before initiation of dialysis. In the PDD group, we separated participants into two groups: continued anti-diabetic drug use after dialysis (pre-existing diabetes dialysis patients continuing medication; PDD-M) and discontinued use (pre-existing diabetes dialysis patients not continuing medication; PDD-NM). The definition of continued medication use was having a record of anti-diabetic drug use for at least 90 days in one year after initiation of dialysis. In the NPDD group, we also identified the patients with new-onset diabetes (incident diabetes after dialysis; IDD) during the follow-up period. New-onset diabetes after entering dialysis was defined as T2DM diagnosed at least three months after dialysis initiation, confirmed by diagnostic codes, and anti-diabetic drug administration history for at least 90 days in one year as previously defined. The remaining patients did not develop diabetes between dialysis initiation and the end of follow-up (non-diabetes dialysis; NDD). The endpoint was all-cause mortality.

Confounding factors such as sex, age, comorbidities, residence, insured premium, and dialysis modality were adjusted accordingly. Individuals with comorbidities were confirmed from their history of comorbidities as diagnosed by ICD coding in at least one admission code, or three or more outpatient codes, before the initiation of dialysis. ICD codes are provided in Additional file 1: Table S1. We collected the number of hospitalizations during the 12 months leading up to dialysis initiation as the indicator of vulnerability. This study also evaluated whether anti-diabetic drug choice affects mortality. We defined the first time of anti-diabetic drug use more than 90 days as the specific drug group and then subgrouped the patients into the: Insulin, SU, Meglitinide, TZD, and DPP-4 inhibitors groups. The use of angiotensin-converting enzyme inhibitors (ACEI), angiotensin II receptor blockers (ARB), and lipid-lowering drugs was also considered as a covariate factor. The details of the Anatomical Therapeutic Chemical (ATC) code of different drugs are provided in Additional file 1: Table S2.

### Data analysis

The SAS software (SAS System for Windows, version 9.2; SAS Institute, Cary, NC) and SPSS statistical software 22.0.0 (IBM Corp., Armonk, NY, USA) were used to perform statistical analyses in this study. Descriptive statistics were used to analyze the baseline demographic data and the distribution of each variable among the study population. Continuous variables were expressed as mean ± SD. Multiple comparisons among the study groups were performed using one-way analysis of variance followed by Bonferroni’s post hoc test. Categorical variables were described as proportions and compared by χ^2^ analysis with Fisher’s exact correction. Overall survival rates were calculated using the Kaplan–Meier method, and the differences in survival was determined by the log-rank test. The stepwise Cox regression method was used for the overall analyses of death in the multivariate model. The entry probability was 0.1 and the removal probability was 0.05. Statistical significance was defined as P < 0.05. The hazard ratios (HR) and their 95 % confidence intervals (CIs) from the Cox regression analyses were used as estimates of relative risk.

## Results

### Clinical characteristics and survival rates of dialysis patients during 2011–2015

**Fig. 1:**
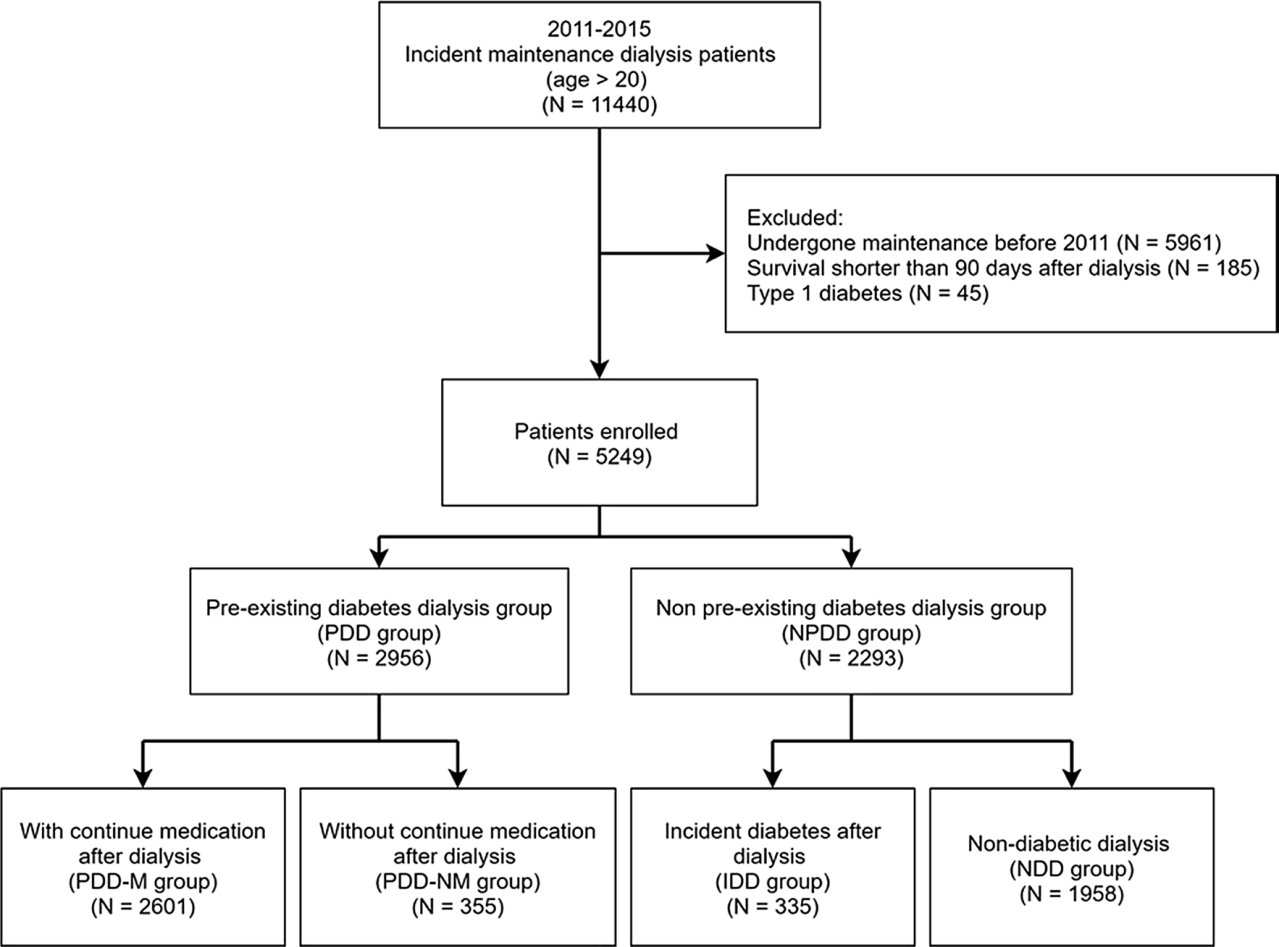
Flow chart of enrolment of participants in the cohort study

We identified a total of 5249 patients on incident maintenance dialysis between 2011 and 2015 from the LHID2010 two million people cohort after evaluating the inclusion and exclusion criteria (Fig. 1). Of these, 2956 (56.3 %) patients were classified into the PDD group and 2293 (43.7 %) into the NPDD group. There were 2601 (88 %) patients in the PDD-M group and 355 (12%) patients in the PDD-NM group. In the NPDD group, there were 335 (14.6 %) patients in the IDD group and 1958 (85.4 %) patients in the NDD group.

Table 1 presents the distribution of demographic characteristics, comorbidities, and mortality outcome in each group: patients in the PDD-NM group were older than the patients in the other groups (P = 0.002); the NDD group had a higher percentage of patients undergoing peritoneal dialysis (P < 0.001); and patients in the PDD group, regardless of PDD-M or PDD-NM, had a higher hospitalization rate more than once in the year before initiation of dialysis, than the patients in the NPDD group (P < 0.001). In addition, the proportion of comorbidities, including hypertension, hyperlipidemia, congestive heart failure, and cerebrovascular accidents was higher in the PDD group than in the NPDD group (P < 0.001). Patients in the PDD-NM group had a lower proportion of ACEI/ARB and lipid-lowering drug usage than other groups (P < 0.001).

**Table 1:** Clinical characteristics and survival rates of dialysis patients during 2011–2015, n = 5249

Variable	PDD		NPDD		P-value
	PDD-M	PDD-NM	IDD	NDD	
	n = 2601 (49.6%)	n = 355 (6.8%)	n = 335 (6.4%)	n = 1958 (37.3%)	
Age, years (mean ± SD)	61 ± 12	64 ± 13	63 ± 14	61 ± 16	0.002
Gender					0.561
Male	1447 (56%)	200 (56%)	179 (53%)	1054 (54%)	
Female	1154 (44%)	155 (44%)	156 (47%)	904 (46%)	
Region					0.058
Northern	1139 (44%)	146 (41%)	165 (49%)	850 (43%)	
Central	617 (24%)	71 (20%)	77 (23%)	477 (24%)	
Southern/Eastern	845 (33%)	138 (39%)	93 (28%)	631 (32%)	
Insured premium (NT$)					0.505
< 15,840	635 (24%)	96 (27%)	96 (29%)	475 (24%)	
15,840–25,000	1187 (46%)	150 (42%)	145 (43%)	874 (45%)	
> 25,000	779 (30%)	109 (31%)	94 (28%)	609 (31%)	
Urbanization					0.775
Urban	1460 (56%)	199 (56%)	179 (53%)	1116 (57%)	
Sub-urban	835 (32%)	108 (30%)	117 (35%)	622 (32%)	
Rural	306 (12%)	48 (14%)	39 (12%)	220 (11%)	
Hospital characteristics (teaching level)					< 0.001
Medical center	726 (28%)	91 (26%)	93 (28%)	668 (34%)	
Region	886 (34%)	119 (33%)	113 (34%)	614 (31%)	
Others	989 (38%)	145 (41%)	129 (38%)	676 (35%)	
Number of admissions during the 12 months leading up to dialysis					< 0.001
0–1	1744 (67%)	212 (60%)	274 (82%)	1574 (80%)	
> 1	857 (33%)	143 (40%)	61 (18%)	384 (20%)	
Dialysis modality					< 0.001
Hemodialysis	2485 (95%)	340 (96%)	316 (94%)	1708 (87%)	
Peritoneal dialysis	116 (5%)	15 (4%)	19 (6%)	250 (13%)	
Comorbidity					
Hypertension	1785 (69%)	270 (76%)	146 (43%)	987 (50%)	< 0.001
Hyperlipidemia	790 (30%)	79 (22%)	58 (17%)	325 (17%)	< 0.001
Gout	233 (9%)	50 (14%)	46 (14%)	338 (17%)	< 0.001
Congestive heart failure	908 (35%)	133 (38%)	83 (25%)	501 (26%)	< 0.001
Cerebrovascular accident	471 (18%)	57 (16%)	41 (12%)	208 (11%)	< 0.001
COPD	185 (7%)	36 (10%)	34 (10%)	166 (9%)	0.053
Chronic liver disease	149 (6%)	33 (9%)	19 (6%)	70 (4%)	< 0.001
Malignancy	199 (8%)	45 (13%)	32 (10%)	212 (11%)	< 0.001
ACEI/ARB use	1555 (60%)	97 (27%)	195 (58%)	878 (45%)	< 0.001
Lipid-lowering drug use	1025 (39%)	45 (13%)	115 (34%)	431 (22%)	< 0.001
Mortality	814 (31%)	166 (47%)	121 (36%)	495 (25%)	< 0.001
Survival, years (mean ± SD)	4.1 ± 1.5	3.0 ± 2.1	4.0 ± 1.5	4.2 ± 1.6	< 0.001

*PDD* pre-existing diabetes dialysis, *NPDD* non-pre-existing diabetes dialysis, *PDD-M* pre-existing diabetes dialysis patients continuing medication, *PDD-NM* pre-existing diabetes dialysis patients not continuing medication, *IDD* incident diabetes after dialysis, *NDD* non-diabetic dialysis, *COPD* chronic obstructive pulmonary disease, *ACEI* angiotensin converting enzyme inhibitors, *ARB * angiotensin II receptor blockers

We conducted a stepwise regression analysis to evaluate the risk factors for mortality in all dialysis patients (Table 2). After multivariable adjustment, the following factors were found to be significant independent predictors of increased mortality: age > 60 years (P < 0.001); diagnosis of diabetes, pre-existing or new-onset (P < 0.001); hospitalization more than once during the 12 months leading up to dialysis initiation (P < 0.001); congestive heart failure (P = 0.012); cerebrovascular accidents (P = 0.001); chronic obstructive pulmonary disease (P < 0.001); chronic liver disease (P = 0.002); and malignancy (P < 0.001). Factors associated with significantly decreased mortality included hypertension (P < 0.001); ACEI or ARB use (P < 0.001); and lipid-lowering drug use (P < 0.001).

**Table 2:** Stepwise regression analysis for all dialysis patients with 5-year survival rate, n = 5249

Variables	Univariate		Multivariate	
	HR (95% CI)	P-value	aHR (95% CI)	P-value
Age > 60 year	2.74 (2.45–3.07)	< 0.001	2.32 (2.08–2.61)	< 0.001
Gender—male	1.06 (0.96–1.18)	0.273	1.00 (0.95–1.05)	0.984
Grouping				
NDD	1		1	
IDD	1.23 (1.09–1.37)	< 0.001	1.35 (1.20–1.52)	< 0.001
PDD-M	1.46 (1.19–1.78)	< 0.001	1.52 (1.25–1.86)	< 0.001
PDD-NM	2.58 (2.16–3.07)	< 0.001	2.12 (1.77–2.53)	< 0.001
Region				
Northern	1			
Central	1.08 (0.95–1.22)	0.231		
Southern/Eastern	0.92 (0.82–1.03)	0.153		
Insured premium (NT$)				
< 15,840	1		1	
15,840–25,000	0.84 (0.74–0.94)	0.003	0.86 (0.75–0.96)	0.010
> 25,000	0.72 (0.63–0.82)	< 0.001	0.85 (0.74–0.97)	0.014
Urbanization				
Urban	1			
Sub-urban	1.11 (0.99–1.24)	0.067		
Rural	1.23 (1.05–1.43)	0.008		
Hospital characteristics				
Medical center	1			
Region/others	1.01 (0.91 -1.13)	0.836		
Number of admissions during the 12 months leading up to dialysis				
0–1	1		1	
> 1	1.87 (1.69–2.07)	< 0.001	1.60 (1.42–1.80)	< 0.001
Dialysis modality				
Peritoneal dialysis	1			
Hemodialysis	1.76 (1.40–2.22)	< 0.001		
Comorbidity				
Hypertension	1.22 (1.10–1.36)	< 0.001	0.78 (0.69–0.88)	< 0.001
Hyperlipidemia	0.78 (0.69–0.88)	< 0.001		
Gout	0.91 (0.78–1.07)	0.251		
Congestive heart failure	1.37 (1.23–1.52)	< 0.001	1.15 (1.03–1.28)	0.012
Cerebrovascular accident	1.51 (1.33–1.71)	< 0.001	1.26 (1.11–1.43)	0.001
COPD	1.84 (1.58–2.14)	< 0.001	1.34 (1.14–1.56)	< 0.001
Chronic liver disease	1.57 (1.30–1.90)	< 0.001	1.36 (1.12–1.66)	0.002
Malignancy	1.81 (1.57–2.09)	< 0.001	1.39 (1.20–1.61)	< 0.001
ACEI/ARB use	0.58 (0.52–0.64)	< 0.001	0.68 (0.62–0.76)	< 0.001
Lipid-lowering drug use	0.50 (0.44–0.57)	< 0.001	0.61 (0.54–0.69)	< 0.001

*HR* hazard ratio, *CI* confidence interval, *aHR* adjusted hazard ratio, *NDD* non-diabetic dialysis, *IDD* incident diabetes after dialysis, *PDD*-NM pre-existing diabetes dialysis patients not continuing medication, *PDD-M* pre-existing diabetes dialysis patients continuing medication, *COPD* chronic obstructive pulmonary disease, *ACEI* angiotensin converting enzyme inhibitors, *ARB* angiotensin II receptor blockers

**Fig. 2:**
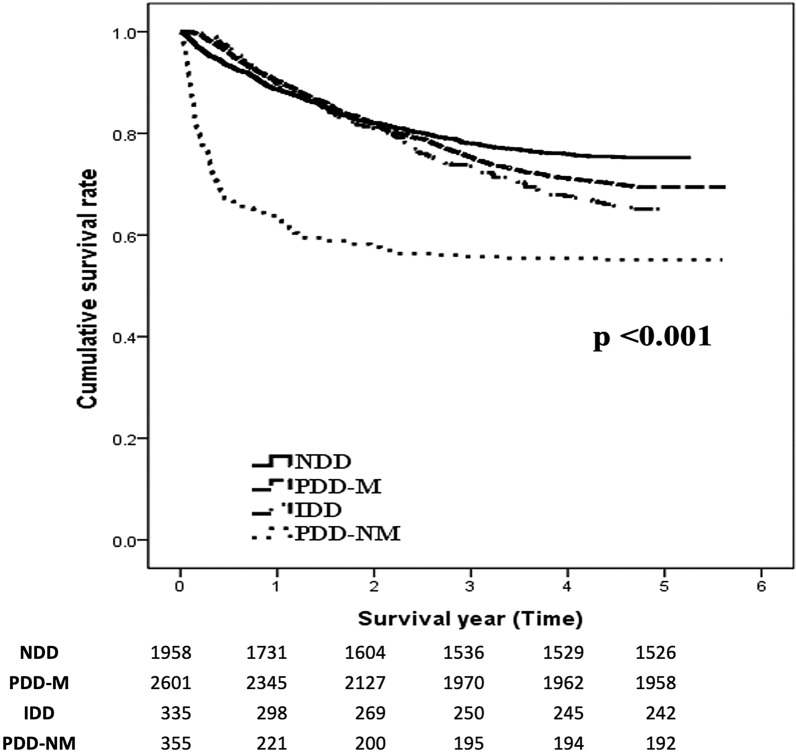
The Kaplan–Meier survival curves between the four groups (PDD-M, PDD-NM, IDD, NDD)

A total of 1596 patients died during the follow-up period, and the Kaplan–Meier survival curve of the four groups is shown in Fig. 2. During the post hoc analysis, the PDD-NM group displayed a significantly higher mortality rate than the IDD, PDD-M, and NDD groups (log-rank test P < 0.001).

### Comparisons in PDD patients during 2011–2015

In Table 3, we also used a stepwise regression model to determine the risk factors of mortality in PDD patients. After multivariable adjustment, factors that were significant independent predictors for increased mortality in the PDD group included: age older than 60 years (P < 0.001), discontinued anti-diabetic drug use (P < 0.001), hospitalization more than once during the 12 months leading up to dialysis initiation (P < 0.001), cerebrovascular accidents (P = 0.002), chronic liver disease (P = 0.001), and malignancy (P = 0.037). By contrast, a decreased risk of mortality was found in patients with hypertension (P = 0.002) or those who were administered ACEI or ARB (P < 0.001) or lipid-lowering drugs (P < 0.001).

**Table 3:** Stepwise regression analysis for pre-existing diabetic dialysis patients with 5-year survival rate, n = 2956

Variables	Univariate		Multivariate	
	HR (95% CI)	P-value	aHR (95% CI)	P-value
Age > 60 year	2.31 (2.01–2.65)	< 0.001	2.05 (1.78–2.35)	< 0.001
Gender—male	1.09 (0.96–1.24)	0.183		
Anti-diabetic drug use				
Non-user	1		1	
OHA	0.44 (0.37–0.53)	< 0.001	0.56 (0.49–0.68)	< 0.001
Insulin	0.52 (0.43–0.63)	< 0.001	0.66 (0.55–0.80)	< 0.001
Region				
Northern	1			
Central	1.01 (0.86–1.19)	0.900		
Southern/Eastern	0.94 (0.81–1.09)	0.398		
Insured premium (NT$)				
< 15,840	1			
15,840–25,000	0.87 (0.75–1.02)	0.084		
> 25,000	0.80 (0.68–0.95)	0.012		
Urbanization				
Urban	1			
Sub-urban	1.09 (0.95–1.25)	0.236		
Rural	1.10 (0.90–1.33)	0.365		
Hospital characteristics				
Medical center	1.03 (0.89–1.19)	0.674		
Region/others	1			
Number of admissions during the 12 months leading up to dialysis				
0–1	1		1	
> 1	1.72 (1.51–1.95)	< 0.001	1.59 (1.38–1.83)	< 0.001
Dialysis modality				
Peritoneal dialysis	1			
Hemodialysis	0.86 (0.64–1.15)	0.306		
Comorbidity				
Hypertension	1.14 (0.98–1.31)	0.070	0.73 (0.67–0.91)	0.002
Hyperlipidemia	0.80 (0.69–0.93)	0.003		
Gout	1.07 (0.86–1.32)	0.562		
Congestive heart failure	1.22 (1.07–1.39)	0.003		
Cerebrovascular accident	1.35 (1.16–1.57)	< 0.001	1.21 (1.03–1.41)	0.020
COPD	1.57 (1.27–1.64)	< 0.001	1.21 (0.98–1.49)	0.087
Chronic liver disease	1.65 (1.32–2.07)	< 0.001	1.48 (1.18–1.86)	0.001
Malignancy	1.65 (1.36–2.01)	< 0.001	1.24 (1.01–1.52)	0.037
ACEI/ARB use	0.59 (0.52–0.67)	< 0.001	0.56 (0.47–0.68)	< 0.001
Lipid-lowering drug use	0.50 (0.43–0.58)	< 0.001	0.66 (0.55–0.80)	< 0.001

*HR* hazard ratio, *CI* confidence interval, *aHR* adjusted hazard ratio, *OHA* oral hypoglycemia agents, *COPD* chronic obstructive pulmonary disease, *ACEI* angiotensin converting enzyme inhibitors, *ARB* angiotensin II receptor blockers

**Fig. 3:**
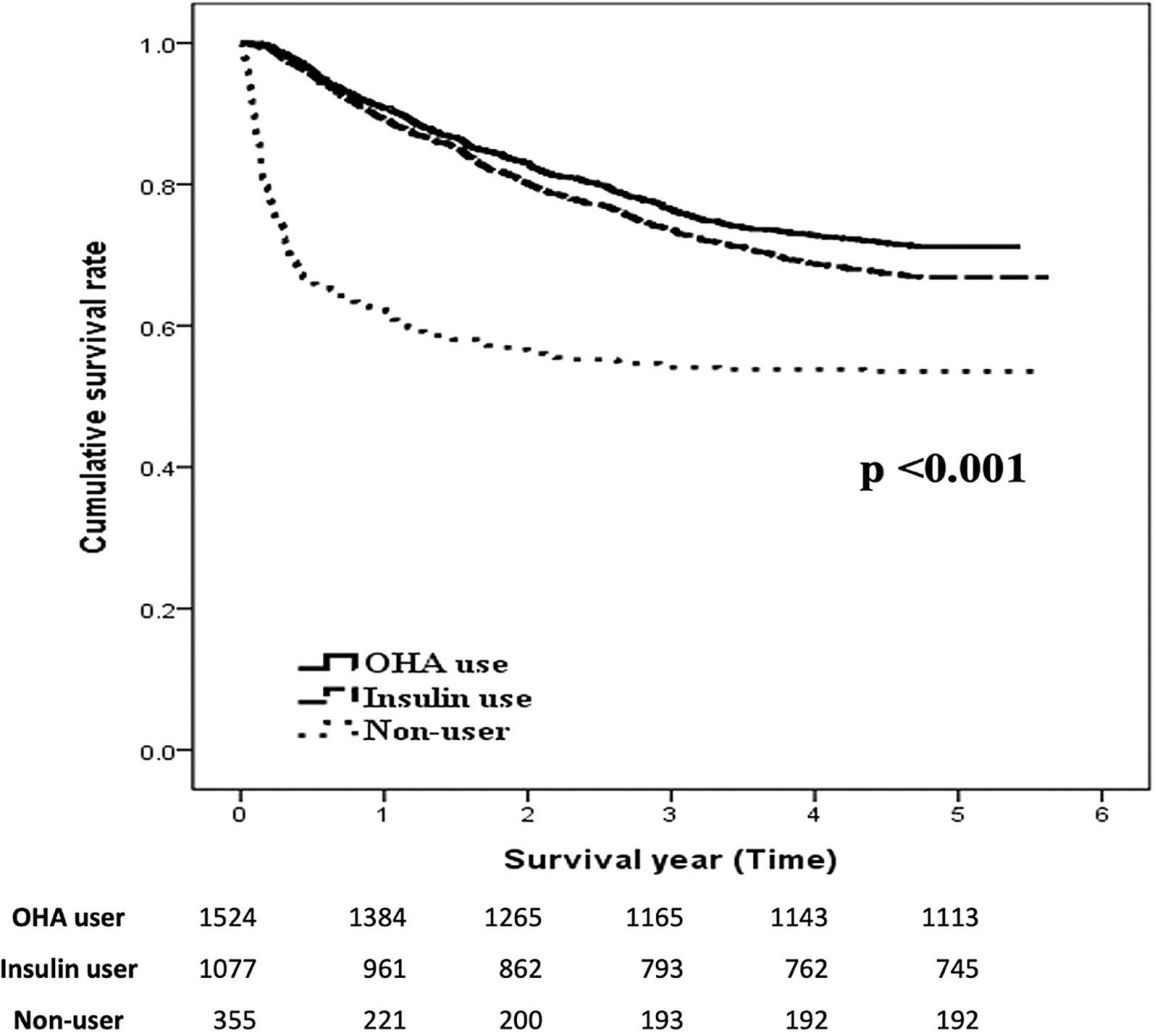
The Kaplan–Meier survival curves for the anti-diabetic drug use subgroups in PDD patients

Figure 3 shows that compared with the PDD-NM group, the PDD-M group had significantly lower risks of all-cause mortality, regardless of insulin (aHR: 0.66; CI 0.55–0.80; P < 0.001) or OHA use (aHR: 0.56; CI 0.49–0.68; P < 0.001). Stratification analysis (Table 4) with different types of anti-diabetic drugs was applied. Results showed better survival benefit than non-users, regardless of the use of SU (aHR: 0.52; CI 0.41–0.67; P < 0.001), meglitinide (aHR: 0.48; CI 0.39–0.60; P < 0.001), TZD (aHR: 0.66; CI 0.65–1.25; P = 0.02), DPP-4 inhibitor (aHR: 0.40; CI 0.32–0.51; P < 0.001), or insulin (aHR: 0.58; CI 0.47–0.68; P < 0.001). Patients using DPP-4 inhibitors seemed to exhibit the greatest improvement.

**Table 4:** Stratification analysis for the survival rate of pre-existing diabetic dialysis patients with different types of anti-diabetic drugs. n = 2956

Variables	aHR (95% CI)	P-value
Anti-diabetic drugs		
Non-user	1	
SU	0.52 (0.41–0.67)	< 0.001
TZD	0.66 (0.65–1.25)	0.020
Meglitinide	0.48 (0.39–0.60)	< 0.001
DPP-4 inhibitors	0.40 (0.32–0.51)	< 0.001
Insulin	0.58 (0.47–0.68)	< 0.001

*aHR* adjusted hazard ratio, *CI* confidence interval, *SU* sulfonylurea, *TZD* thiazolidinedione, *DPP-4* dipeptidyl-peptidase IV

### Analysis of patients with IDD during 2011–2015

As shown in Table 5, factors that increased the risk of mortality in the IDD group included age older than 60 years (P < 0.001), insulin use (compared with OHA use; P = 0.008), hospitalization more than once during the 12 months leading up to dialysis initiation (P = 0.031), cerebrovascular accidents (P = 0.003), and chronic obstructive pulmonary disease (P = 0.009). After stepwise multivariate regression analysis, significant independent predictors for increased mortality in the IDD group were age older than 60 years (P < 0.001), cerebrovascular accidents (P = 0.003), and chronic obstructive pulmonary disease (P = 0.039). Patients with hyperlipidemia had a lower risk (P = 0.014). Medication with insulin or OHA had no statistically significant difference in mortality in the overall IDD group. However, further stratification analysis (Table 6) indicated that OHA users had a better survival rate than insulin users in the older (aHR: 1.74; CI 1.09–2.77; P = 0.02) and male subgroups (aHR: 1.76; CI 0.99–3.11; P = 0.05).

**Table 5:** Stepwise regression analysis for incident diabetic patients after dialysis with 5-year survival rate, n = 335

Variables	Univariate		Multivariate	
	HR (95% CI)	P-value	aHR (95% CI)	P-value
Age > 60 years	2.52 (1.67–3.79)	< 0.001	2.35 (1.56–3.55)	< 0.001
Gender—male	1.40 (0.98–2.00)	0.068		
Anti-diabetic drug use				
OHA	1			
Insulin	1.63 (1.14–2.33)	0.008	1.41 (0.98–2.02)	0.068
Region				
Northern	1			
Central	1.23 (0.80–1.89)	0.900		
Southern/Eastern	0.94 (0.63–1.52)	0.398		
Insured premium (NT$)				
< 15,840	1			
15,840–25,000	0.78 (0.51–1.20)	0.254		
> 25,000	0.87 (0.55–1.38)	0.543		
Urbanization				
Urban	1			
Sub-urban	0.75 (0.50–1.12)	0.157		
Rural	0.84 (0.47–1.62)	0.565		
Hospital characteristics				
Medical center	1.19 (0.81–1.76)	0.378		
Region/others	1			
Number of admissions during the 12 months leading up to dialysis				
0–1	1			
> 1	1.59 (1.05–2.43)	0.031		
Dialysis modality				
Peritoneal dialysis	1			
Hemodialysis	1.24 (0.54–2.81)	0.614		
Comorbidity				
Hypertension	1.22 (0.85–1.75)	0.275		
Hyperlipidemia	0.43 (0.23–0.81)	0.008	0.45 (0.24–0.85)	0.014
Gout	0.85 (0.50–1.46)	0.557		
Congestive heart failure	1.27 (0.85–1.89)	0.239		
Cerebrovascular accident	2.03 (1.28–3.22)	0.003	2.04 (1.27–3.29)	0.003
COPD	1.92 (1.18–3.14)	0.009	1.68 (1.03–2.76)	0.039
Chronic liver disease	1.00 (0.47–2.15)	0.998		
Malignancy	1.65 (0.98–2.80)	0.061		
ACEI/ARB use	0.68 (0.48–0.97)	0.033		
Lipid-lowering drugs use	0.50 (0.33–0.76)	0.001	0.66 (0.43–1.02)	0.060

*HR* hazard ratio, *CI* confidence interval, *aHR* adjusted hazard ratio, *OHA* oral hypoglycemia agents, *COPD* chronic obstructive pulmonary disease, *ACEI* angiotensin converting enzyme inhibitors, *ARB* angiotensin II receptor blockers

**Table 6:** Stratification analysis for incident diabetic patients after dialysis with 5-year survival rate, n = 335

Variables	aHR (95% CI)	P-value
Age		
> 60 years		
OHA	1	
Insulin	1.74 (1.09–2.77)	0.020
≤ 60 years		
OHA	1	
Insulin	1.08 (0.46–2.57)	0.855
Sex		
Male		
OHA	1	
Insulin	1.76 (0.99–3.11)	0.050
Female		
OHA	1	
Insulin	1.09 (0.62–1.95)	0.758

*aHR* adjusted hazard ratio, *OHA* oral hypoglycemia agents

## Discussion

The purpose of this retrospective cohort study using an NHI database was to investigate whether concomitant diabetes or anti-diabetic drug use affects the prognosis of dialysis patients. This epidemiological study provides a preliminary understanding of the importance of diabetes disease control and drug selection in this group. Here, three issues were raised for discussion. First, dialysis patients with diabetes had higher mortality than those without, regardless of pre-existing diabetes at initiation of dialysis or new-onset diabetes after entering dialysis. Second, for the PDD group, those who continued to use anti-diabetic medications after entering dialysis had a lower risk of death. In the subgroup analysis, patients using SU, meglitinide, TZD, DPP-4 inhibitor, or insulin all showed significantly better outcomes compared with non-users. Third, the choice of either insulin or OHA had no significant effect on overall survival in the new-onset diabetes after dialysis group. However, initial OHA use was associated with a better prognosis in the male and older than 60 years subgroups.

In our study, we confirmed that T2DM had a significant impact on the prognosis of patients undergoing dialysis. The previous study showed the survival of hemodialyzed diabetic patients was not inferior to non-diabetics [13]. On the contrary, our study result revealed that patients undergoing dialysis with pre-existing diabetes had a 32 % higher mortality rate than NDD patients, and patients with new-onset diabetes after dialysis also had a 44 % increased death risk. This finding was compatible with other study results in different countries [3, 14, 15]. Our results also confirmed that diabetes mellitus is independently associated with the risk of developing hypertension, cerebrovascular accidents, and congestive heart failure. Because diabetes has such a huge impact on the dialysis population, it should be actively treated and controlled.

One of the challenges regarding the management of diabetic dialysis patients is the uncertainty of blood sugar control goals. In the DOPPS study, the HbA1C level strongly predicted mortality in hemodialysis patients with type 1 or type 2 diabetes [16]. The association between HbA1C and mortality was U-shaped, and the mortality was lowest at HbA1C 7–7.9 %. The KDOQI guideline also emphasized that in those dialysis patients with significant comorbidities or limited life expectancy, HbA1C control was preferably greater than 7, higher than in normal people [17]. However, another study also showed the effect of glycemic control in dialysis patients differed according to age and dialysis type and should be carefully planned and monitored [18]. Therefore, we suggested that glycemic control should be individualized assessment and management. Many important issues still need to be considered when we treat diabetic dialysis patients, including the accuracy of HbA1C measurement in the renal failure population [19, 20], the burn-out diabetes phenomenon [21], and whether strict blood sugar control would be too late to improve cardiovascular benefits [22].

Few diabetic patients may experience spontaneous resolution of hyperglycemia status and fluctuating HbA1C levels to as low as < 6 % after entering dialysis. Some experts [9, 23] defined this phenomenon as “burn-out diabetes”. Multiple factors may contribute to this condition, including malnutrition, protein-energy wasting, prolongation of insulin half-life due to decreased clearance, reduction of renal gluconeogenesis, accumulation of uremic toxin, and glucose removal by hemodialysis [7]. Anti-diabetic medications like insulin or other OHAs were suggested to be discontinued to minimize the risk of hypoglycemia in this situation [24]. Although etiological analysis is lacking, we believe the burn-out phenomenon is one of the most important reasons for withdrawal of anti-diabetic medication after initiation of dialysis. However, the adverse effects of insulin resistance in such patients are continuing progression despite near-normal HbA1C levels. In our study, patients who discontinued medication were older, had a higher prevalence of congestive heart failure, malignancy, and chronic liver disease, and there was less RAS blockade usage, all of which have been described as obvious determinants of mortality in dialyzed individuals. However, after these factors and the number of hospitalizations in the past year were adjusted for, continued anti-diabetic drug use was still an independent predictor of favorable survival rate. Thus, we speculate that anti-diabetic medication use has possible additional benefits to directly improve outcomes, such as decreased insulin resistance, improved endothelial function, and anti-inflammatory effects [25, 26]. The actual mechanisms of this association still need more research to explore.

Our subgroup analysis also showed that patients using SU, meglitinide, TZD, DPP-4 inhibitor, or insulin all had significantly better outcomes compared with non-users. Balancing anti-diabetic medication administration and hypoglycemic risk is an important issue. In this situation, the use of DPP-4 inhibitors appears to be more reasonable and valuable. Compatible with our finding, Chan et al. indicated that DPP-4 inhibitor users in diabetic dialysis patients had a lower risk of all-cause mortality than non-users [27]. The survival benefits may be attributable to significantly fewer ischemic stroke events among DPP-4 inhibitor users due to neuroprotective effects. DPP-4 inhibitors have a greater reduction in HbA1C than in fasting blood sugar, which can lower the possible side effect of hypoglycemia and reduce blood glucose variability [28, 29]. In addition, DPP-4 inhibitors resulted in fewer weight gain side effects, more protective of pancreatic beta-cell function and volume, and better tolerability [30]. An additional benefit for dialysis patients includes reduced inter-dialytic weight accumulation [31]. We suggest that DPP-4 inhibitors are an appropriate and suitable choice for diabetic dialysis patients who are at high risk of hypoglycemia, regarding monotherapy or add-on treatment.

According to a previous report [32], the prevalence of new-onset diabetes after dialysis was around 12.7 % within a ten-year follow-up period; the incidence was significantly associated with female sex, age, and baseline comorbidity. The 5-year incidence in our study was 14.6 %. We further analyzed the relationship between different anti-diabetic medication use in patients with new-onset diabetes after dialysis and mortality, with the results indicating that initial treatment with insulin or other OHAs did not affect the outcome. Stratification analysis revealed that OHA users had a better survival rate than insulin users only in the old and male subgroups. These groups are traditionally susceptible to cardiovascular disease and cerebrovascular disease [33]. Some studies showed OHA has a better protection effect than insulin use [34, 35]. Another possible explanation is that patients who started on insulin injection treatment usually had poor blood sugar control, implying an increased susceptibility to large vessel complications [36]. Our results are worthy of further larger randomized trials to verify their significance.

There are some limitations to our study. First, this observational cohort study using the NHIRD could only show associations and not explain direct causality. However, epidemiological studies can provide multivariate comparisons and reveal specific impact factors, then there can be further prospective studies or randomized controlled trials to confirm causality. Second, because all definitions of variables were based on clinical physicians’ diagnosis and coding, misidentification may result in bias. Third, the NHIRD does not provide personal history, lifestyle information, and laboratory results—such as HbA1C or serial glucose level—which are potential confounding factors for this study, and this is a major inherent limitation in the study. Fourth, we did not explore the relationship between the reasons for drug withdrawal and the cause of death in our study. Last, we grouped drug type based on the first drug used more than 90-days and we did not evaluate long-term medication compliance. This problem requires a more precise randomized clinical trial to build more solid evidence.

## Conclusions

Our study used an NHI database to conduct large-scale epidemiological research on the incident maintenance dialysis group. We found that dialysis patients with diabetes certainly had a higher mortality rate, regardless of whether diabetes was pre-existing or new-onset. Our novel finding was that continued use of anti-diabetic drugs after dialysis and choice of medication may affect clinical outcomes. Further rigorous studies are needed to confirm the trends and associations discovered by our research.

## Supplementary Information

**Additional file 1: Table S1.** ICD-9 and ICD-10 codes used to identify comorbidities. **Table S2.** Anatomic therapeutic chemical (ATC) classification codes for select medications.

## Data Availability

The data used to support the findings of this study are available from the corresponding author upon reasonable request.

## Abbreviations

ACEI: Angiotensin-converting enzyme inhibitors
ARB: Angiotensin II receptor blockers
DPP-4: Dipeptidyl-peptidase IV
ESRD: End-stage renal disease
HbA1C: Hemoglobin A1C
HWDC: Health and Welfare Data Science Center
IDD: Incident diabetes after dialysis.
KDOQI: Kidney Disease Outcomes Quality Initiative
LHID2010: Longitudinal Health Insurance Database 2010
NDD: Non-diabetes dialysis
NPDD: Non-pre-existing diabetes dialysis group
NHI: National Health Insurance
NHIRD: National Health Insurance Research Database
OHA: Oral hypoglycemic agents
PDD: Pre-existing diabetes dialysis group
PDD-M: Pre-existing diabetes dialysis patients continuing medication
PDD-NM: Pre-existing diabetes dialysis patients not continuing medication
SU: Sulfonylureas
T2DM: Type 2 diabetes mellitus
TZD: Thiazolidinediones
