# Supplementary material for: Effect of anti-diabetic drugs in dialysis patients with diabetes: a nationwide retrospective cohort study
Source: Cardiovasc Diabetol. 2021 Sep 8;20:179. doi: 10.1186/s12933-021-01364-w (PMC8424811; doi:10.1186/s12933-021-01364-w)
Supplement: Supplementary file 1 — Additional file 1: Table S1. ICD-9 and ICD-10 codes used to identify comorbidities. Table S2. Anatomic therapeutic chemical (ATC) classification codes for select medications. [file 12933_2021_1364_MOESM1_ESM.docx]

**Additional file 1**

**Additional file 1 Table of Contents**

|  | Page number |
| --- | --- |
| Additional file 1: Table S1 ICD-9 and ICD-10 codes used to identify comorbidities | 2 |
| Additional file 1: Table S2 Anatomic therapeutic chemical (ATC) classification codes for select medications | 3 |

**Additional file 1: Table S1.** ICD-9 and ICD-10 codes used to identify comorbidities

| Diagnosis | ICD-9-CM code | ICD-10-CM code |
| --- | --- | --- |
| Hypertension | 401–405 | I10–I16 |
| Hyperlipidaemia | 272 | E77–E78, E75.3, E71.30, E88.89 |
| Gout | 274 | M10 |
| Congestive heart failure | 398.91, 422, 425, 428, 402, 404 | I09.81, I40–I43, I50, I11, I13 |
| Cerebrovascular accidents | 430–438 | I60–I63, I65-I69, G45–G46 |
| Chronic obstructive pulmonary disease | 491–494, 496, 510 | J41–J45, J47 |
| Chronic liver disease | 571 | K70–K76.9 |
| Malignancy | 140–208 | C00–C96 |

ICD-9-CM: International Classification of Diseases, Ninth, Clinical Modifications; ICD-10-CM: International Classification of Diseases, Ninth and Tenth Revision, Clinical Modifications

**Additional file 1: Table S2.** Anatomic therapeutic chemical (ATC) classification codes for select medications

| Class of medication | ATC codes |
| --- | --- |
| Sulfonylurea | A10BB |
| Thiazolidinedione | A10BG |
| Meglitinide | A10BX02, A10BX03, A10BX05, A10BX08 |
| Dipeptidyl-peptidase IV inhibitors | A10BH |
| Insulin | A10AB, A10AC, A10AD, A10AE, A10AF |
| Angiotensin converting enzyme inhibitors | C09AA, C09BA, C09BB, C09BX |
| Angiotensin II receptor blockers | C09CA, C09DA, C09DB, C09DX |
| Lipid-lowering agents | C10AA, C10AB, C10AC, C10AD, C10AX, C10BA, C10BX |
